# RETALT: review of technologies and overview of design changes

**DOI:** 10.1007/s12567-022-00458-9

**Published:** 2022-06-28

**Authors:** Ansgar Marwege, Ali Gülhan, Josef Klevanski, Christian Hantz, Sebastian Karl, Mariasole Laureti, Gabriele De Zaiacomo, Jan Vos, Matthew Jevons, Christoph Thies, Anett Krammer, Marc Lichtenberger, João Carvalho, Sofia Paixão

**Affiliations:** 1 DLR Institute of Aerodynamics and Flow Technology, Supersonic and Hypersonic Technologies Department, Linder Hoehe, 51147 Cologne, Germany; 2 DLR Institute of Aerodynamics and Flow Technology, Spacecraft Department, Bunsenstrasse 10, 37073 Göttingen, Germany; 3 Flight Systems Business Unit DEIMOS Space S.L.U., Atmospheric Flight Competence Center, Ronda de Poniente, 19. Edificio Fiteni VI, portal 2, 28760 Tres Cantos (Madrid), Spain; 4 CFS Engineering, EPFL Innovation Park, Batiment-A, 1015 Lausannes, Switzerland; 5 Future Programs, MT Aerospace AG, Franz-Josef-Strauß-Straße 5, 86153 Augsburg, Germany; 6 Almatech Space and Naval Engineering, EPFL Innovation Park, Batiment-D, 1015 Lausanne, Switzerland; 7 Amorim Cork Composites, Rua de Meladas 260, 4536 902 Mozelos VFR, Portugal

**Keywords:** Retro propulsion, Vertical take-off vertical landing technologies

## Abstract

RETALT (RETro propulsion Assisted Landing Technologies) is a project funded in the frame of the European Union Horizon 2020 program, that is studying critical key technologies for the vertical landing of launcher configurations with the aid of retro propulsion. In particular Aerodynamics, Aerothermodynamics, Flight Dynamics and Guidance Navigation and Control (GNC), Structures, Mechanisms, Thrust Vector Control and Thermal Protection Systems are investigated in detail in the project. This paper provides an overview of the technological achievements in these different technological areas, with emphasis on the interaction between them. Design changes made to the RETALT1 configuration are laid out in detail. The novel approach of using interstage segments as aerodynamic control surfaces proved to be challenging from the aerodynamics, flight dynamics, mechanical and structural points of view. For this reason, planar fins were introduced as aerodynamic control surfaces in the new base line configuration for RETALT1. The paper concludes with a summary of future steps to be made in the RETALT project to reach the targeted Technology Readiness Level (TRL) of the different key technologies.

## Introduction

Since the successes of Blue Origin with the New Shepard and SpaceX with the Falcon 9 and Falcon Heavy launch vehicles, Vertical take-off and Vertical Landing of launch vehicles in different payload classes can be considered as the state-of-the-art in the USA. In Europe the know-how for the implementation of this concept is still being build up. In projects like Ariane Next [1] and ENTAIN [2], system studies and trade-offs were performed. EAGLE [3], Frog [4] and DTV [5] were projects considering small scale demonstrators for the development of GNC algorithms. The RETPRO [6] project specifically targets the validation of CFD tools and wind tunnel experiments for the generation of aerodynamic data for reusable launchers. And projects as Callisto [7] and Themis [8] focus on the building of large-scale demonstrators representative of a launcher first stage to study the necessary technologies as a whole.

The project RETALT (RETro propulsion Assisted Landing Technologies) is funded by the European Union’s Horizon 2020 research and innovation framework program under grant agreement No 821890. It started in March 2019 targeting the investigation of specific key technologies of vertical descent and landing. An overview of the project was presented in Ref. [9]. The outcome of the project is not in a single area of research as for the EAGLE, Frog, DTV and RETPRO projects or toward a large-scale demonstrator as in Callisto or Themis. The RETALT project aims to generate new profound knowledge, computational and experimental expertise and data in the key disciplines needed to develop vertically landing reusable launcher configurations using retro propulsion. In addition, the project will also provide a better understanding of the interplay between these key disciplines.

The key technologies studied in RETALT are: Aerodynamics, Aerothermodynamics, Flight Dynamics and Guidance Navigation and Control (GNC), Structures, Mechanisms, Thrust Vector Control and Thermal Protection Systems. The targeted Technology Readiness Level to be reached at the end of the project (August 2022) is 5 for Aerodynamics, Aerothermodynamics, Structures, Mechanisms and Thermal Protection Systems, and 3 for Flight Dynamics and Guidance, Navigation and Control (GNC).

To meet the overall project objective of understanding the key technologies for vertically landing launchers with the aid of retro propulsion, two reference configurations are studied, namely (for details refer to Ref. [9]):

- RETALT1: A heavy lift launcher configuration able to bring a payload of up to 14 t into the Geostationary Transfer Orbit (GTO)
- RETALT2: A smaller Single Stage To Orbit (SSTO) configuration able to bring a payload of 500 kg into Low Earth Orbits (LEO)

One main advantage of vertical descent and landing using retro propulsion is that the load direction is similar to the ascent configuration. Furthermore, the main deceleration is performed by the engines. This means that only a small number of additional parts and limited changes to an expendable launch vehicle are necessary to implement this reusability concept. This is the principal reason that these configurations promise large cost savings, as the reusability can be obtained with a relatively small increase in system complexity.

Due to its higher relevance for the use of reusability for European launchers in the midterm, the focus of the project was on the RETALT1 configuration, which was studied in more detail than RETALT2. The design of RETALT1 evolved in the course of the project due to input from the different disciplines investigated.

This paper is laid out as follows. First an overview of the reference configurations is given. Then, the design evolution of RETALT1 is discussed that includes a detailed reasoning of the design choices. This is followed by a summary of the technological achievements in the project so far, highlighting the interaction between the various involved disciplines. The paper will conclude with a summary and an outlook of future work to be performed.

## Overview of the RETALT reference configurations

**Fig. 1:**
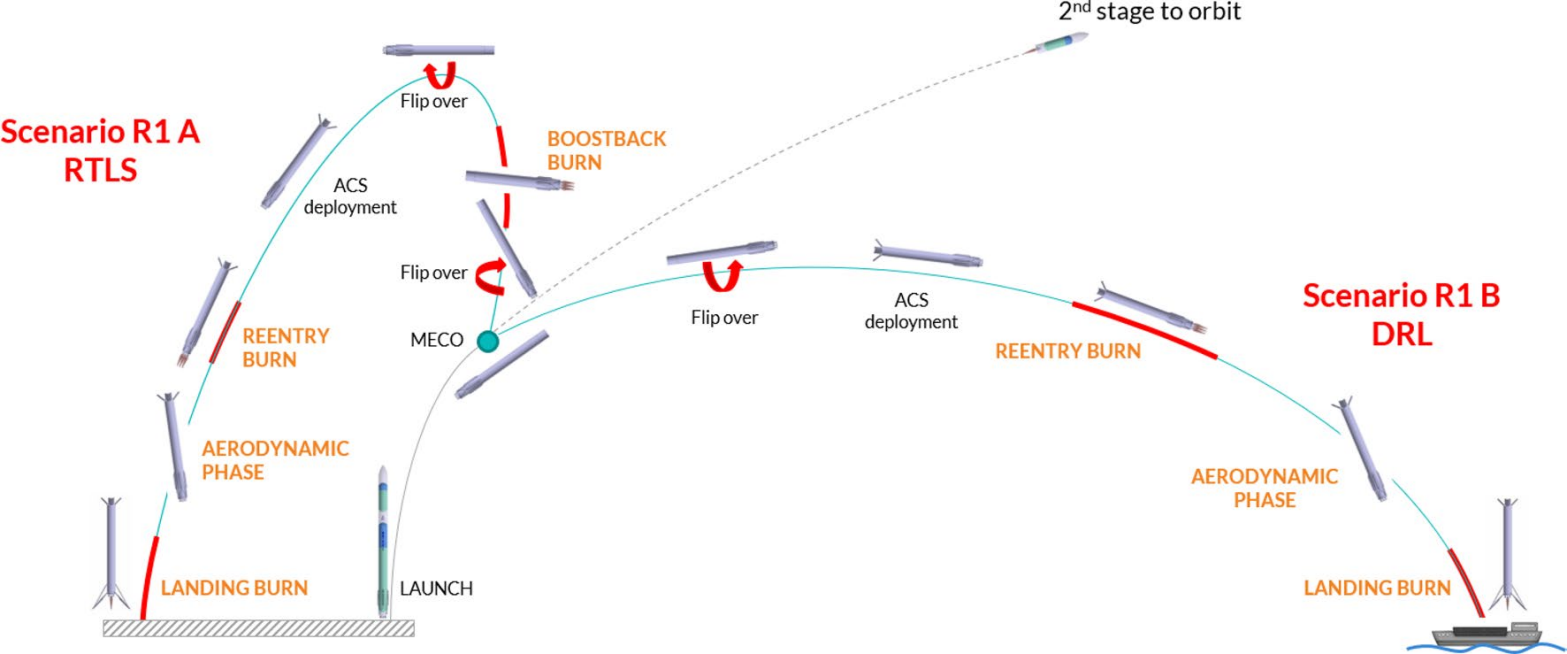
RETALT1 return mission concept [10]

**Fig. 2:**
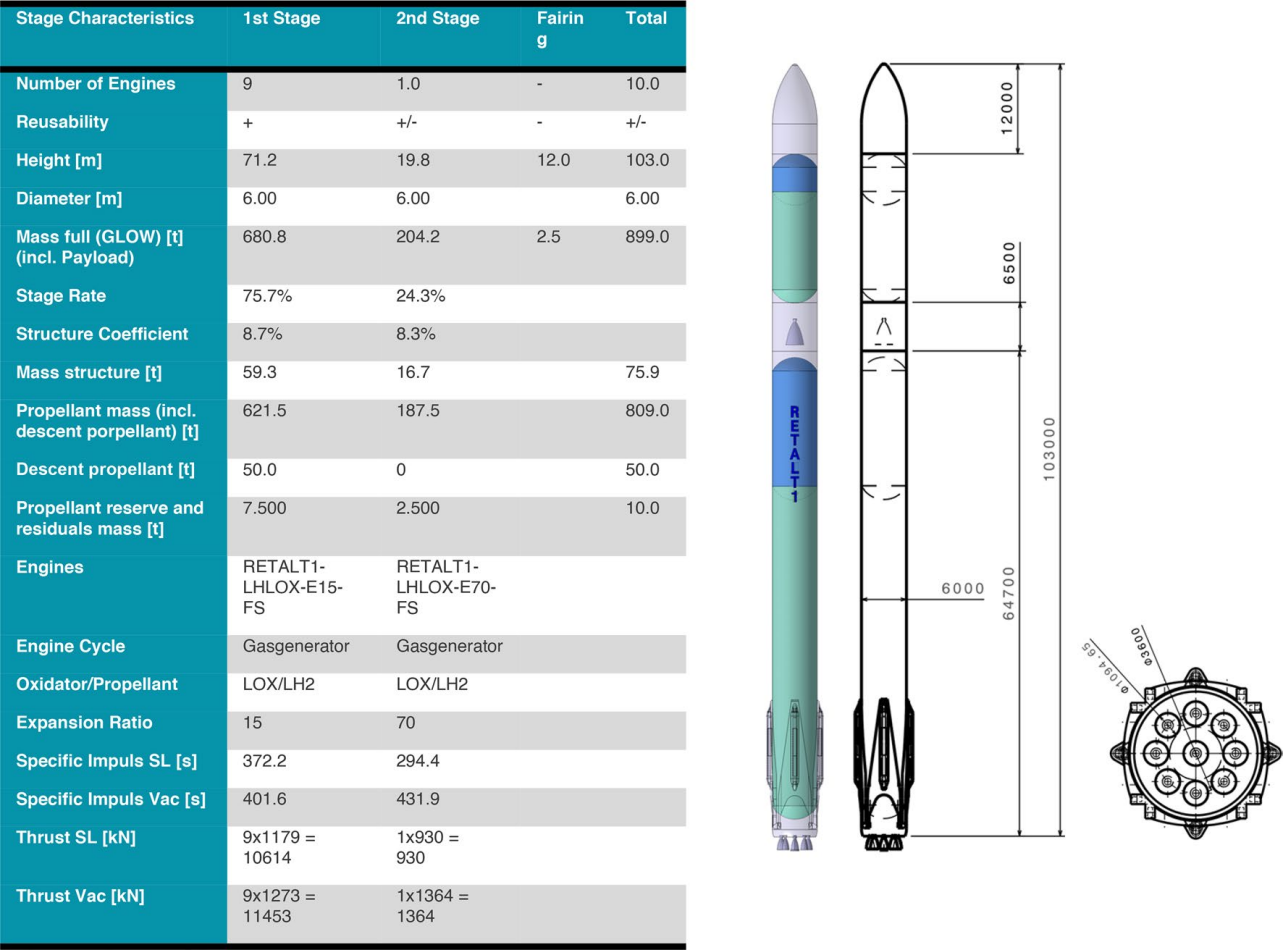
Main characteristics of the RETALT1 configuration as presented in Ref. [9]

The mission concepts for the RETALT1 configuration are shown in Fig. 1. The main characteristics of RETALT1 are summarized in Fig. 2. Similar to the Falcon 9 by SpaceX it can either Return To Launch Site (RTLS) or perform a Down Range Landing (DRL) on a seagoing platform or barge. In the case of the Down Range Landing, after the Main Engine Cut Off (MECO) and the stage separation, the first stage is flipped over, such that its engines are pointing into the flight direction and the Aerodynamic Control Surfaces (ACS) are deployed. Then while reentering the atmosphere, a re-entry burn is performed with three active engines to decelerate the vehicle, and reduce the heat loads and dynamic pressure during the following aerodynamic phase in which the engines are not active and the configuration is flying purely aerodynamically. In the final phase a pinpoint landing is performed with the final landing burn. In the case of the Return To Launch Site scenario, a flip over maneuver and a boostback burn are performed after MECO to direct the stage back to the launch site. The other phases are the same as for the DRL scenario.

**Fig. 3:**
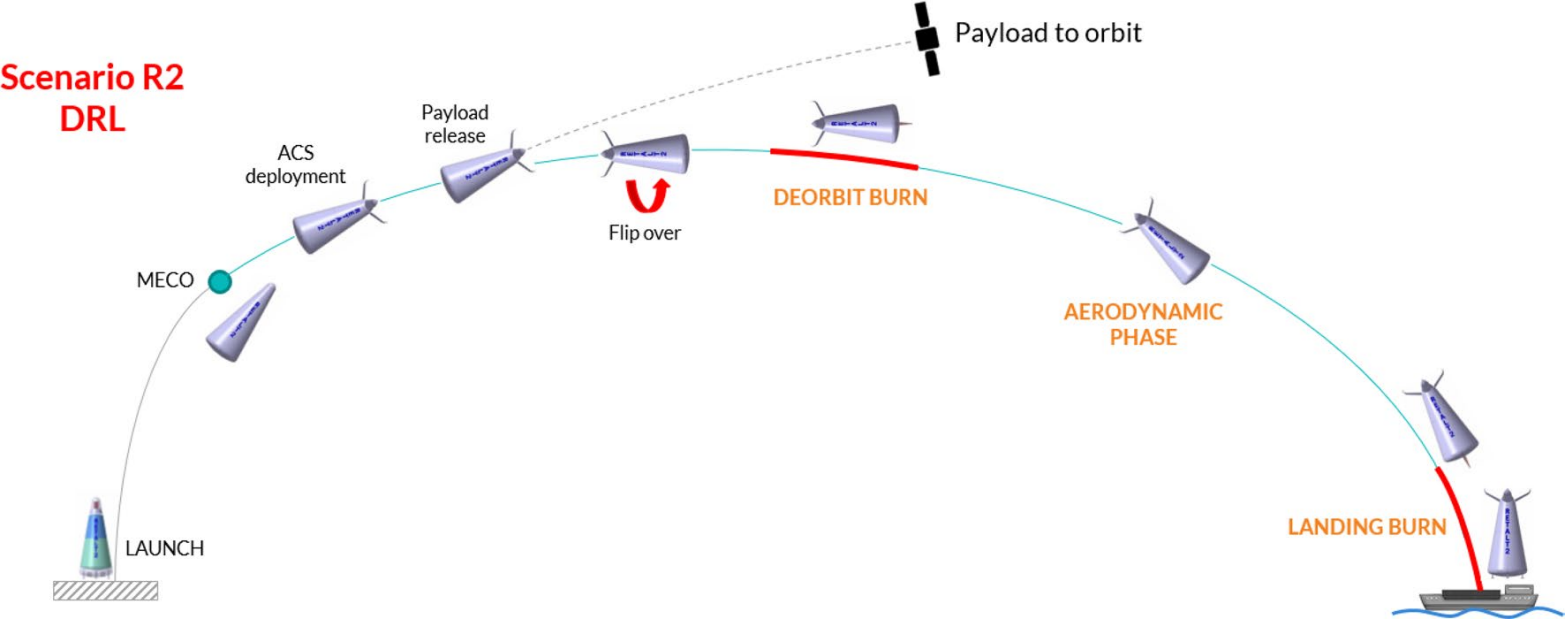
RETALT2 return mission concept

**Fig. 4:**
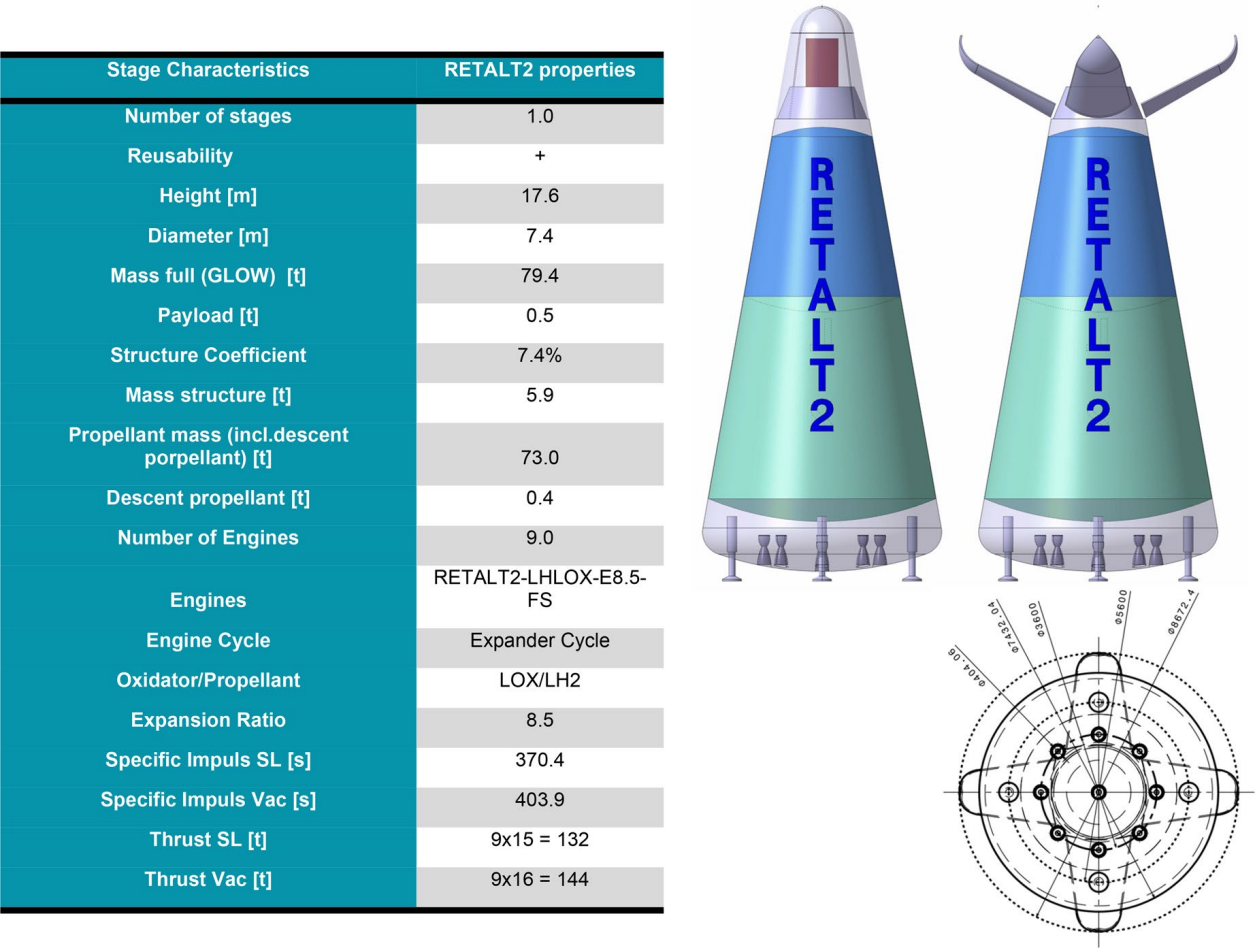
Main characteristics of the RETALT2 configuration as presented in Ref. [9]

The mission concept of RETALT2 is shown in Fig. 3. Its main characteristics are summarized in Fig. 4. The concept of RETALT2 is to decelerate the vehicle mainly by aerodynamic drag. This is why the shape of RETALT2 is inspired by the shape of a re-entry capsule in the rear area, for an increased surface area during descent with large radii to reduce heat loads. After the MECO, the fairing is opened and the payload is released. Then a flip over maneuver and a deorbit burn are performed. The re-entry is performed purely aerodynamically without a re-entry burn. After the aerodynamic phase a pinpoint landing is performed on a seagoing platform.

While the RETALT2 configuration is more academic, the RETALT1 configuration is closely related to existing European technologies and its implementation in European reusable launchers could be performed in the next 5–10 years.

## Design changes of the RETALT1 configuration

**Fig. 5:**
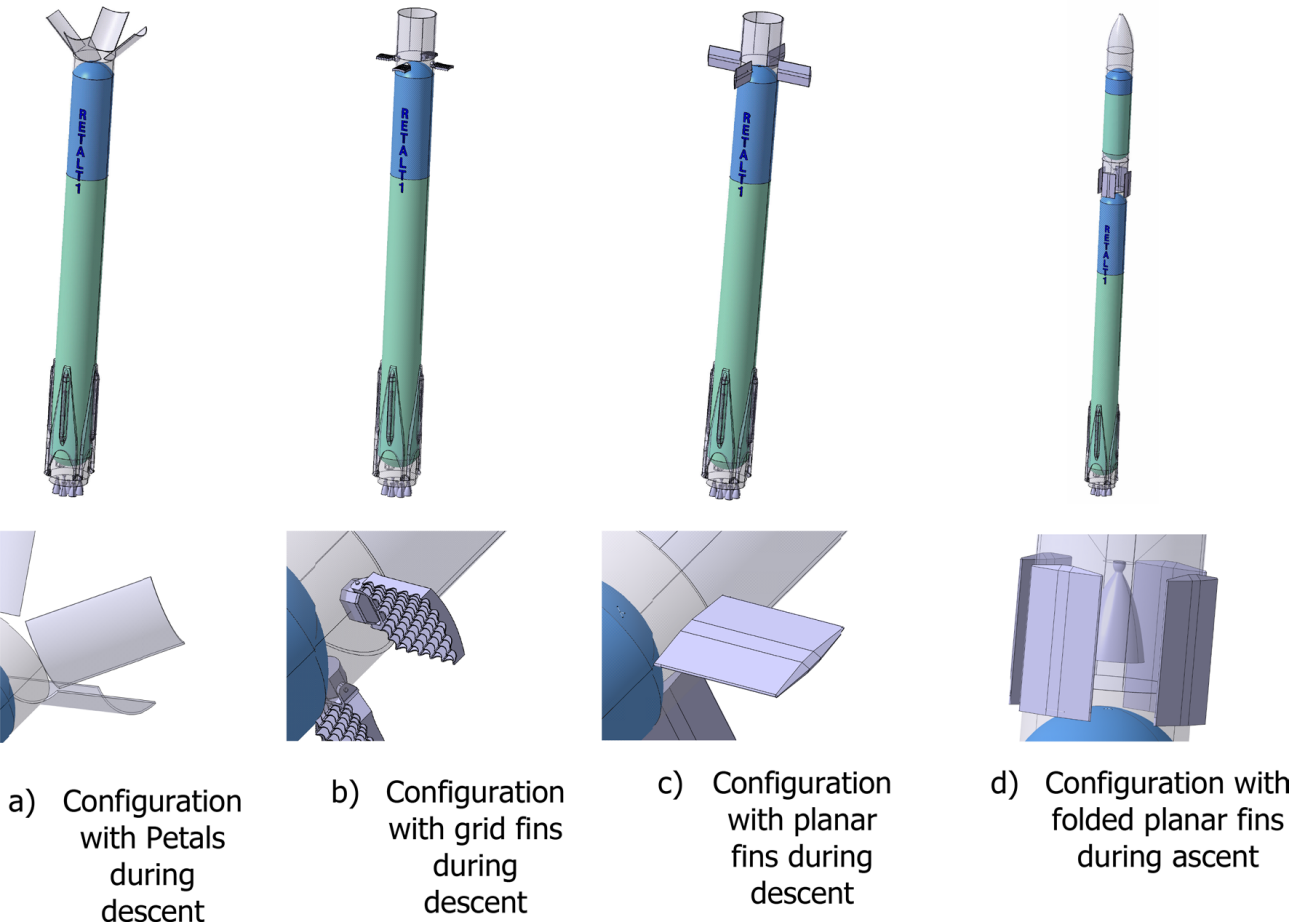
RETALT1 configurations of aerodynamic control surfaces

The original RETALT1 configuration included a novel concept for Aerodynamic Control Surfaces shown in Fig. 5a. The interstage seg ments (also called petals) were intended to be used for the trim of the vehicle, and for the generation of additional aerodynamic drag for the deceleration of the vehicle in the aerodynamic phase. This configuration should be compared to a configuration with grid fins (Fig. 5b).

A study by DEIMOS Space presented in Ref. [10] concluded that the angle of attack at which the vehicle should be trimmable during the aerodynamic phase should be up to 10°. Based on this condition and based on the reference trajectories presented in Ref. [9], different variants of the interstage segments for the aerodynamic control were sized by DLR. This, in turn, delivered load boundary conditions for the structures and for the actuation of the aerodynamic control surfaces. However, load analyses by MT Aerospace showed that the structural mass of the segments were unfeasibly high. Relaxing the requirement that the interstage segments should generate aerodynamic drag reduced the structural mass, but not to a sufficient extend. In addition, when looking to the actuators needed to deploy the interstage segments, it was shown by Almatech that such actuators largely exceeded realistic mass budgets (see Sect. 4).

Finally, the decision was taken to change the original baseline configuration of RETALT1. Instead of using interstage segments for the aerodynamic control, the interstage remains fully closed and planar fins are used for the aerodynamic control, see Fig. 5c. The fins are folded in the ascent phase (see Fig. 5d), which is a design that was also used in the CALLISTO project (see for example Ref. [11]). Compared to the CALLISTO project, the design of the fins was adapted to the specific requirements of the RETALT1 configuration, e.g. the fins are folded “upwards” pointing to the tip rather than pointing “downwards” in the direction of the base of the vehicle. As for the petals, the planar fins where sized to ensure the trimmability of the vehicle at 10° angle of attack during the aerodynamic phase. Even though the base line configuration of RETALT1 was changed to using planar fins for aerodynamic control, the study of the petal configuration was continued to obtain a better understanding of the use of the interstage segments (petals) for aerodynamic control, and to assess the feasibility of this concept for other launcher configurations.

**Fig. 6:**
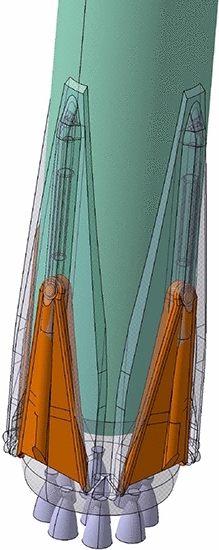
Comparison of initial design of landing legs with updated design (orange) after structural and mechanical design phase

In addition to the Aerodynamic Control Surfaces (ACS), also the landing legs were modified after a detailed structural and mechanical design performed by MT Aerospace and Almatech. Figure 6 shows the updated design in orange compared to the initial design. A detailed discussion of the landing leg structural design can be found in Ref. [12].

## Design change: from petals to planar fins

### Considerations on petals

As mentioned in Sect. 3, it was decided to investigate planar fins as aerodynamic control surfaces for RETALT1. The motivation for the change in the baseline configuration is discussed in this section.

The initial design for RETALT1 was based on the use of the complete interstage as aerodynamic control surfaces. The length of the ACS is thus simply the complete length of the interstage, which is 6.5 m (see Fig. 2). As a first assumption to generate data for the computations of the reference trajectories, they were deflected by 45°. The forces on the petals from this configuration were provided to MT Aerospace and Almatech for a first sizing of the structure and the actuators. These analyses showed that the forces and moments on the petals would result in unfeasibly high structural and actuator masses. For this reason, a more detailed analysis of the petals was performed.

A first analysis was made by DEIMOS to assess how much aerodynamic drag should be provided by the petals [10]. It was shown that a higher aerodynamic drag results in lower dynamic pressures and heat loads on the vehicle in the aerodynamic phase. However, this study also showed that the drag does not necessarily need to be provided by aerodynamic breaking devices. It could also be generated by flying the vehicle at an angle of attack of approximately 10°. This yields comparable drag forces, but relaxes the requirements of the loads on the structures and actuators of the petals.

This study led to a new question: can the RETALT1 configuration be trimmed using the interstage segments (petals) at an angle of attack of 10°, and if affirmative, how do they need to be sized to enable this? This question can be answered as follows.

**Fig. 7:**
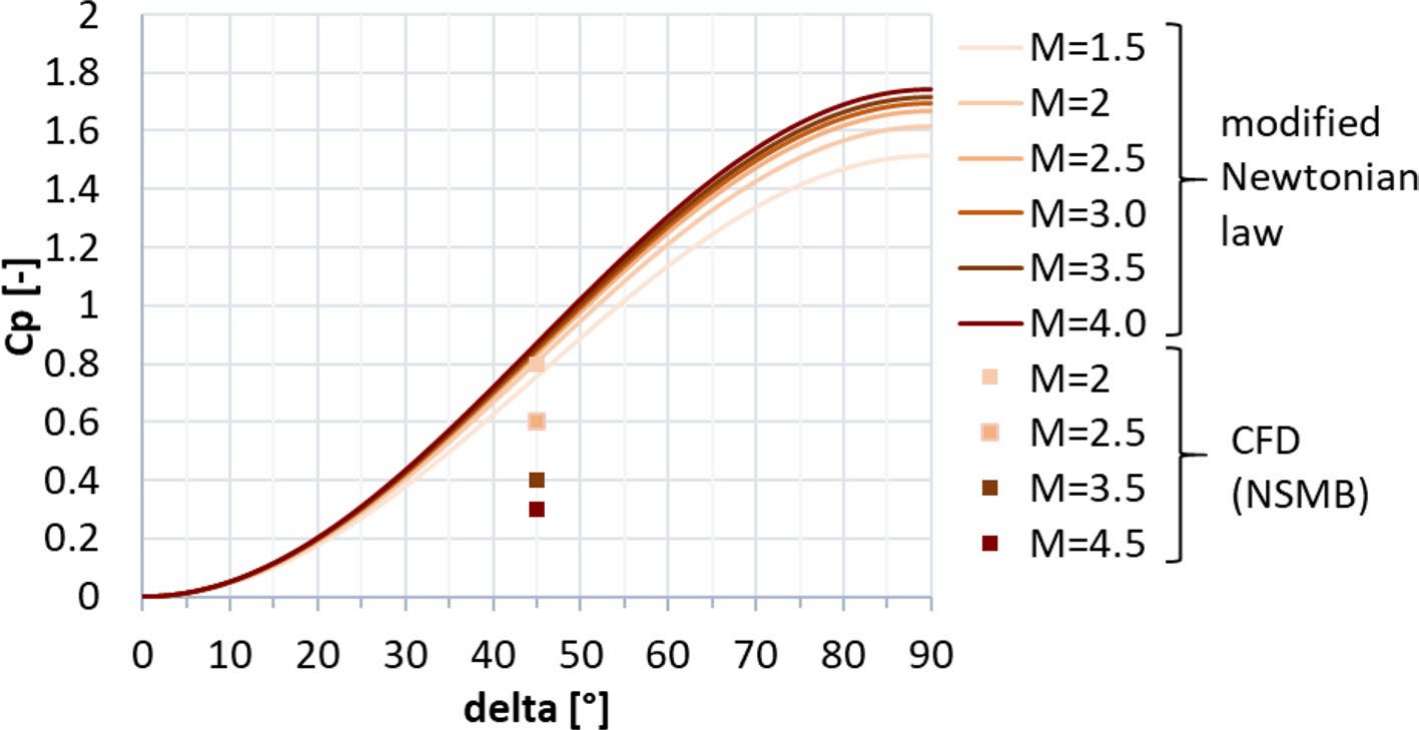
Maximum pressure coefficient on windward side of the petal estimated with the modified Newtonian law and computed with CFD

**Fig. 8:**
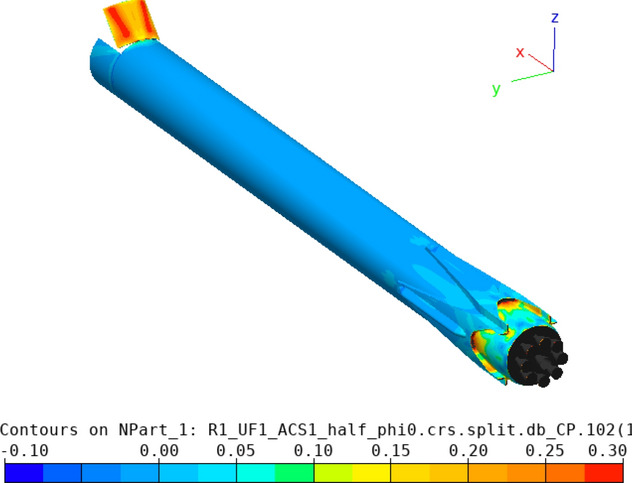
Distribution of pressure coefficient (Cp) from Navier–Stokes CFD computation of RETALT1 with one deflected petal at 45° at Mach 4.5 and at an angle of attack of 0°

**Fig. 9:**
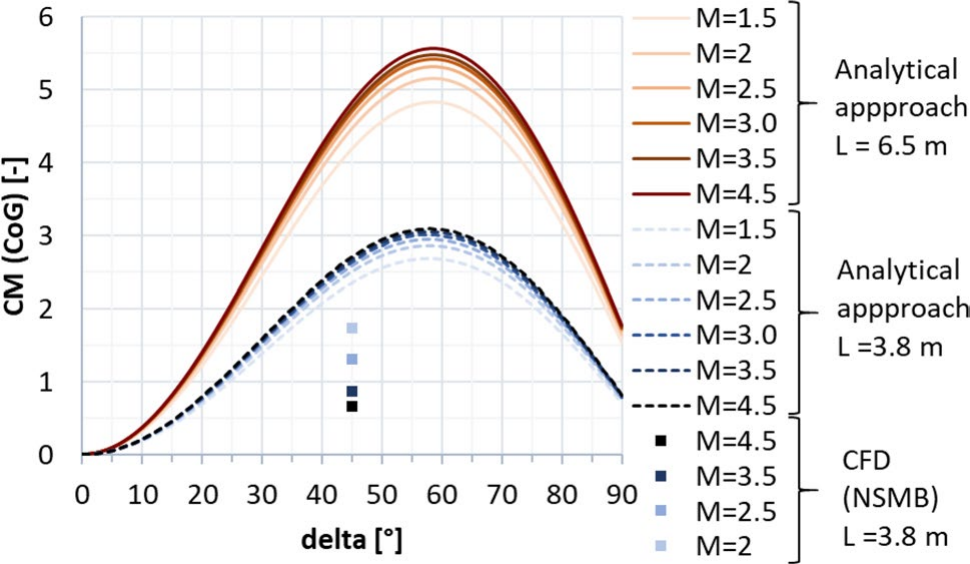
Analytical and CFD solutions for the moment coefficient around the center of gravity

**Fig. 10:**
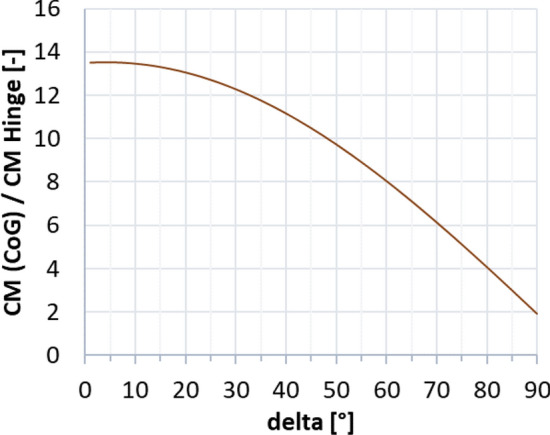
Moment coefficient divided by the hinge moment coefficient. (following from the analytical approach, with a petal length of 6.5 m)

**Fig. 11:**
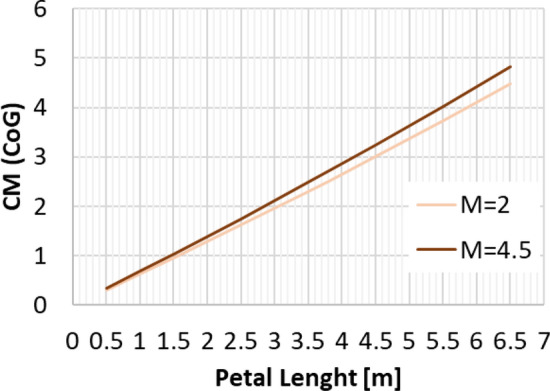
Moment coefficient for a deflection of 45° for different petal length

The highest dynamic pressure is expected in the supersonic regime between Mach 4.5 and 2 [10]. Therefore, the petals were initially sized for this regime. The trim moment shall be generated by the deflection of one single petal, with all the other petals remaining in the closed position. As a first approximation, the pressure on the windward side of the deflected petal was estimated using the modified Newtonian law [13], where the Mach number at the location of the aerodynamic control surfaces was estimated with the blast wave analogy [14]. This methodology was discussed in detail in Ref. [15]. The pressure on the leeward side was assumed to be the static free stream pressure. This permits to calculate the trim moment and moment coefficient with the center of gravity (CoG) as the moment reference point, CM (CoG). The maximum pressure coefficient (Cp) resulting on the windward side of the petal with the modified Newtonian law is shown in Fig. 7 (solid lines). It is compared to Navier–Stokes CFD computations by CFSE with the NSMB flow solver (see dots in Fig. 7.). One exemplary computation is shown in Fig. 8 for Mach 4.5. Due to complex flow phenomena upstream of the petals, as described in Refs. [15, 16], the modified Newtonian law overestimates the maximum pressure. The modified Newtonian law dictates that the pressure coefficient follows the sine squared of the deflection $\delta$. The solid lines in Fig. 9 show the moment coefficient around the center of gravity for a length of the petals of *L* = 6.5 m (the complete length of the interstage segment, see Fig. 2) where the pressure was estimated with the Newtonian law. Several observations can be made. The moment coefficient increases with the deflection angle $\delta$, however, it reaches a maximum and then decreases again. This behavior results from the fact, that, while the pressure coefficient increases with the deflection $\delta$, the lever arm decreases with increasing deflection. Hence an optimum of the generated moment coefficient is reached at $\delta$ = 59°. As for the mass optimization of the actuators, the hinge moment shall be kept low, in Fig. 10 the generated moment coefficient per hinge moment is plotted. This ratio is independent of the Mach number, as the moments results from the same forces but are just shifted in the reference point the dependence is purely geometric. The dependence shows that with increasing deflection angle the hinge moment necessary to generate a certain moment coefficient increases. Therefore, keeping the deflection angle at 45°, to keep the hinge moment low and at the same time reach high moment coefficients seems reasonable. The results shown in Fig. 11 are obtained for a constant deflection angle of 45° and a varying length of the petal for Mach 2 and 4.5.

**Fig. 12:**
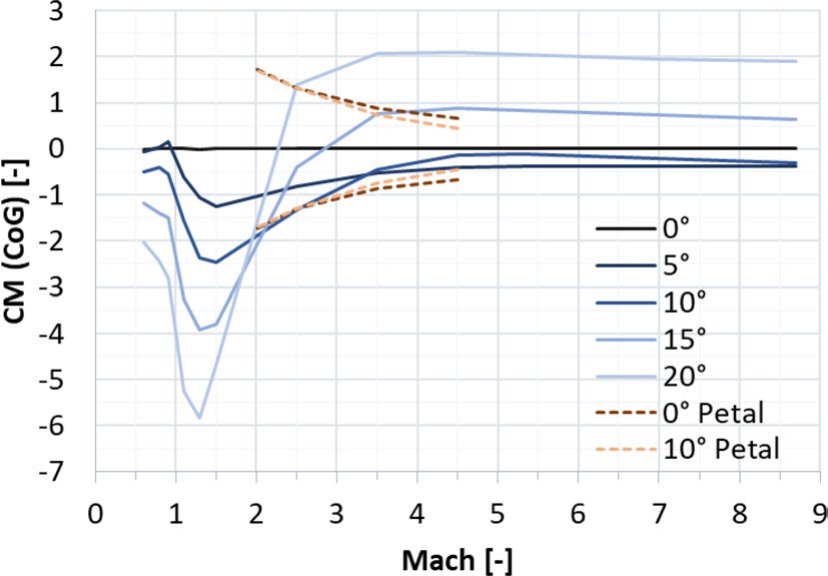
Solid lines: Moment coefficients of the cylindrical body of RETALT1 around the center of gravity (from Euler computations). Dashed lines: moment around center of gravity generated by petals (*L* = 3.8 m, ${\varvec{\delta}}=45^\circ$; from Navier–Stokes computations)

From a first aerodynamic database based on Euler computations with the DLR flow solver TAU, the moment coefficients around the center of gravity of the cylindrical body of RETALT1 without petals were extracted, they are shown in Fig. 12 as solid lines. The largest moment coefficient to be compensated to trim the vehicle at 10° for all Mach numbers is about 2 for Mach 2.0. Therefore, the length of 6.5 m of the petal can be reduced as Fig. 11 shows that already a petal with a length of 3.8 m can reach this value including some safety margin.

**Fig. 13:**
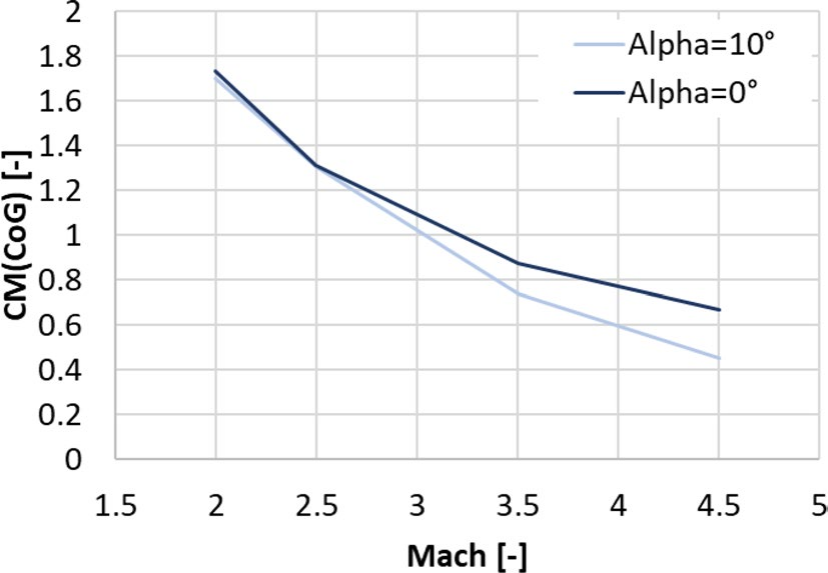
Moment coefficients generated by the petals around the CoG at angles of attack 0° and 10° (*L* = 3.8 m, ${\varvec{\delta}}=45^\circ$)

In Fig. 9 the analytical solution of the petals with 3.8 m length is shown by the dashed lines. These results were cross-checked with Navier–Stokes CFD computations by CFSE with the NSMB flow solver (see dots in Fig. 9). It can be seen that the analytical approach overestimates the generated moment coefficient quite largely. This could be expected as the pressure coefficient on the windward side is overestimated and due to the simplification made that the same pressure is acting on the complete petal while the pressure is actually varying over the petal surface due to their curvature (see Fig. 8). Furthermore, the formulation of the modified Newtonian law leads to the results that the moment coefficient increases with larger Mach numbers, while it actually decreases. An increase of the generated moment coefficient with decreasing Mach numbers is favorable as larger moment coefficients are needed for lower Mach numbers to trim the vehicle. The moment coefficient of the petals versus the Mach number is plotted in Fig. 13 at 0° and at 10° angle of attack. It can be seen that especially at low Mach numbers the influence of the angle of attack is minor. In Fig. 12, the dashed lines show the CM (CoG) of the petal generated at 0° and 10° angle of attack. It can be seen that the deflection of one petal can theoretically generate enough moment to trim the vehicle at angles of attack of 10° in the Mach number range of 2.0–4.5. However, later wind tunnel experiments in the Trisonic Wind Tunnel Cologne (TMK) at the DLR in Cologne showed that even though the moment coefficient is high enough to trim the vehicle, no statically stable configuration around the angle of attack of 0° is reached with the deflection of only one petal [15].

**Fig. 14:**
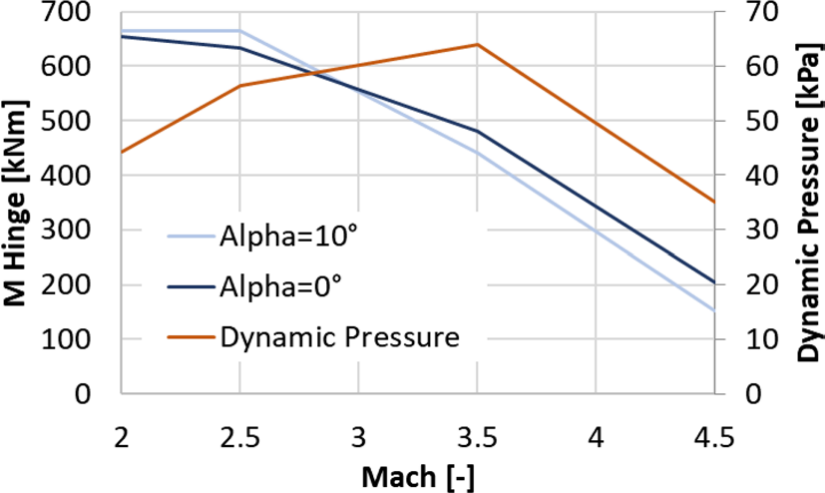
Hinge Moments generated by the petals (*L* = 3.8 m, ${\varvec{\delta}}=45^\circ$)

Figure 14 shows the hinge moment extracted from the CFD computations. For Mach 2.0 they reach a maximum of 654.4 kNm and 665.0 kNm for 0° and 10° angles of attack, respectively. The forces on the petals are then 344.4 kN and 350 kN, respectively. It shall be noted that due to the strong Mach number dependence of the moment coefficient and the force coefficient, the largest hinge moment does not occur at the maximum dynamic pressure, which is also shown in Fig. 14, and that appears at around Mach 3.5.

To size the structure of the petals parametrically, an analytical approach was chosen based on beam bending theory. As such, the petal was represented as two beams, one in the circumferential direction and one in the longitudinal direction. It was assumed that the petal would be made from a stiffened panel design and thus the beam properties were defined from the skin and stiffener geometries and stiffener count along the respective directions. The pressure loading was then simplified as a line load along each idealized beam. The stiffeners are assumed to be blade stiffeners, which are readily viable to be implemented in CFRP applications. Since the approach above is linear elastic a margin of 1.5 was applied to the reserve factor, to ensure to provide margins in the design. The results of the structural sizing of the petals is shown in Table 1. At a realistic value of the stroke at 160 mm, the combined mass of the petals alone exceeds the baseline mass allowable (which was estimated to be 4 t for all petals) and provides little mass allowance for the actuation (if a stretch mass allowance is used).

**Table 1:** Resulting structural petal mass for different actuator attachment heights

Actuator attachment height* [mm]	Skin t [mm]	Circumferential			Longitudinal			Mass [kg]	
		*h* [mm]	*t* [mm]	RF	*h* [mm]	*t* [mm]	RF	Petal	4 Petals
160	3	125	8	1.52	150	8	1.5	859	4124
320	3	125	8	1.52	145	8	1.5	840	4030
480	3	125	8	1.52	135	8	1.5	801	3843
640	3	125	8	1.52	130	8	1.5	781	3750
800	3	125	8	1.52	125	8	1.52	762	3657
960	3	125	8	1.52	120	8	1.54	742	3563

Regarding the actuator requirements to drive a petal, these are very demanding and not catered for by aerospace actuators currently available on the market. These are mostly linear actuators used for thrust vectoring. During the study, electromechanical and electro-hydrostatic actuators were considered with up to 350 kN stall load and 200 mm stroke, and no suitable actuator was identified. Besides the need to support very high loads, stroke and speed limitations were also explored: smaller maximum force actuators can be used if the actuation lever arm is increased; however, this can only be done at the expense of larger stroke and mechanism components being exposed to the environment generated during descent. The petal concept requires development of large custom actuators, as well as appropriately strengthened joints and supporting mechanism structure to cope with the loads. This, in turn, implies increased vehicle mass as well as additional development time and cost.

In conclusion, the resulting hinge moments and forces on the petals are very large resulting in large actuators and structures, while planar fins provide larger moments and forces with less mass effort (see also Ref. [17]). Therefore, the baseline configuration was changed to planar fins. Note that in Ref. [17] the hinge moment of the petal was reported to be 890.3 kNm, which is the value resulting from the assumption of a maximum dynamic pressure of 100 kPa, which was foreseen as limit during descent.

Even though the baseline configuration was changed, detailed wind tunnel experiments of the petals were performed in the Trisonic Wind Tunnel Cologne (TMK), at the DLR in Cologne. They were tested at subsonic and supersonic Mach numbers. The results showed, that not only the structural and actuator mass could be challenging for this configuration. But also trimmability and stability with the petal concept is critical. The results of the wind tunnel experiments are presented in Ref. [15].

### Planar fins design

The new baseline configuration of RETALT1 with planar fins is shown in Fig. 5c and d. The design of the planar fins will be summarized shortly in the following.

**Fig. 15:**
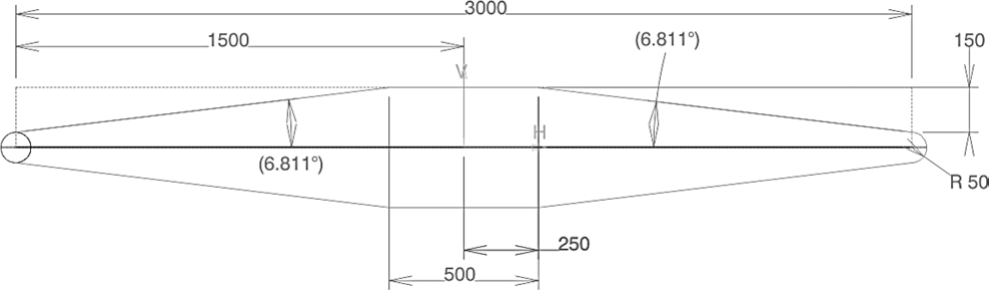
Hexagon profile of the planar fins of the RETALT1 configurations (dimensions in mm)

For the design of the planar fins a hexagon profile was selected which is shown in Fig. 15. The chord of the fins was chosen to be equal to the radius of the launcher, ensuring a tight folding of the fins in the ascent phase (see Fig. 5d). The fins were folded upwards as, this way the attachment point, when the fins are folded is in the area of the interstage rather than on the tanks. One of the requirements of RETALT1 is that the aerothermodynamic heating in the aerodynamic phase shall be kept low, and as a result a large leading-edge radius was chosen for the fin profile [18].

The sizing in the supersonic regime was performed modeling the planar fins with shock and expansion relations, where, as for the petals, the Mach number at the location of the fins was estimated with the blast wave analogy (as described in Ref. [15]). In the subsonic regime the sizing was performed with a modification of the lifting slope equations for thin low-aspect-ratio straight wings proposed in Ref. [19].

**Fig. 16:**
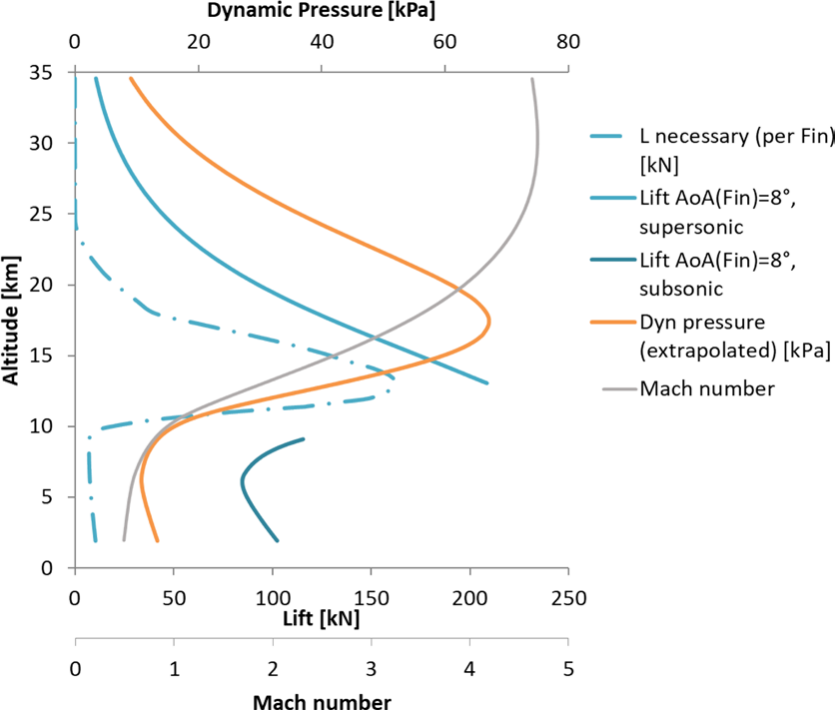
Lift generated by the planar fins for the reference trajectory and necessary lift to trim the vehicle at 10° AoA

**Fig. 17:**
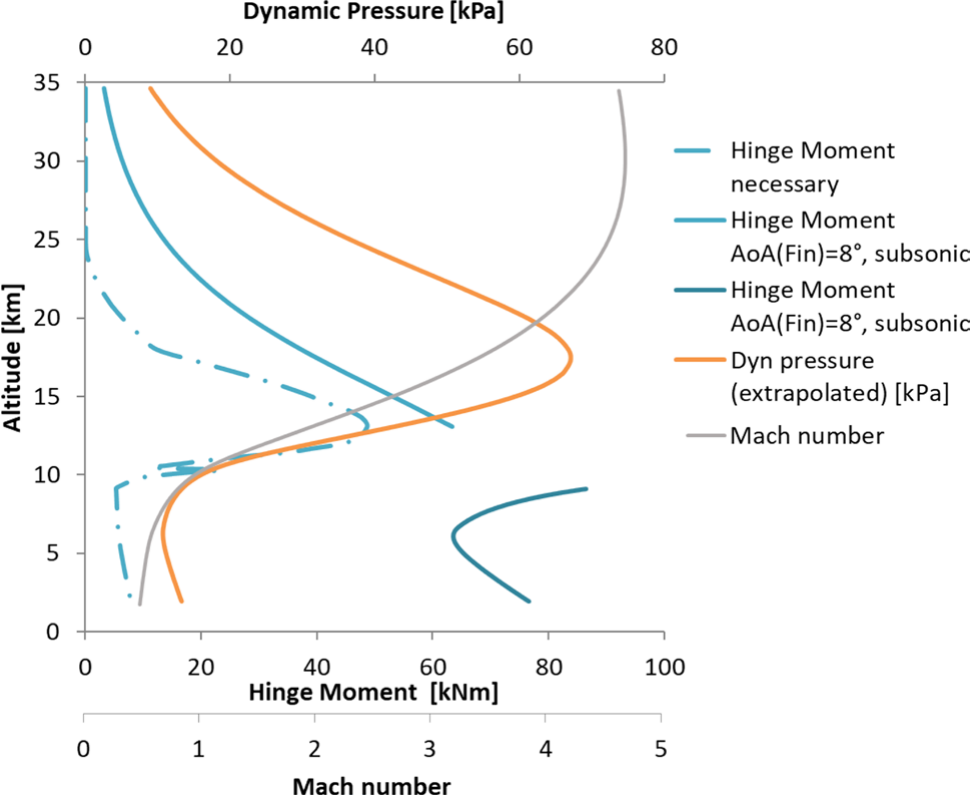
Hing Moment generated by the planar fins for the reference trajectory and necessary hinge moment to trim the vehicle at 10° AoA

The span of the fins was defined such that the fins produce the necessary lift to trim the vehicle at 10° angle of attack throughout the trajectory. This is shown in Fig. 16. The effective angle of attack of the fins was limited to 8° as for larger angles of attack flow separation can be expected for such thin airfoils in the subsonic regime [19]. The span of the fin which resulted from this analysis is 5 m (for one fin). The lift and drag forces generated by the fins create a hinge moment. With the center of pressure known for the supersonic regime and assumed to be at ¼ of the chord in the subsonic regime (as can be expected for thin airfoils in the subsonic regime), the hinge moment can be estimated. Figure 17 shows the hinge moment resulting for a constant angle of attack of the fins of 8° and the hinge moment which would follow if the fins would be deflected such to just generate the necessary lift to trim the vehicle (the necessary hinge moment). It can be seen that the hinge moment would be overestimated if a constant effective angle of attack of 8° would be assumed for the fins.

As a result of this analysis the maximum hinge moment to be provided by the actuators amounts to 100 kNm (in comparison to the approximately 670 kNm necessary for the petals). The maximum normal force of the fins was set to 300 kN. This new fin design was the baseline for the aerodynamic control surface demonstrator and the deployment and actuation developed for it, presented in Ref. [17]. Furthermore, a mission analysis was performed for this configuration in Ref. [10]. It was also tested in the Trisonic Wind Tunnel Cologne at DLR Cologne.

## Technological advancements in the RETALT project

As in the rest of the world, the COVID-19 pandemic influenced the progress of the work in the RETALT project. In the first 2.5 years of the project the foundation of the common research activities was laid out and different hardware was built and partially tested, among them wind tunnel models, a scale model of the aerodynamic control surfaces and TPS materials. This section provides a short summary of the technological advancements in the different key technology areas.

### Aerodynamics and aerothermodynamics

Initial studies were performed for the sizing of the interstage segments as aerodynamic control surfaces and for the generation of aerodynamic drag. Due to the change of the baseline configuration of RETALT1, it was necessary to make additional studies for the design of the planar fins. These analyses were performed by DLR and CFSE by combining analytical design approaches, fast Euler Computations and high fidelity Navier–Stokes CFD.

Once the design of the RETALT1 configuration with planar fins was frozen an Aerodynamic Data Base (AEDB) and an Aerothermodynamic Database (ATDB) were generated by DLR and CFSE for the following reasons: (1) to obtain a thorough understanding of the aerodynamic characteristics of the retro propulsive descent and landing, (2) to serve as input for the Flight Dynamics and GNC developments in the project, (3) to characterize the occurring heat loads for critical structures and for the TPS development. Furthermore, CFD computations were performed to compare the performance of the planar fins against the interstage segments and grid fins. The results are presented in Ref. [16]. The aerothermal results are given in Ref. [18].

The RETALT1 configuration was furthermore tested in the Trisonic Wind Tunnel Cologne (TMK) to study its aerodynamic properties in the aerodynamic flight phase with no active engines. Detailed results of the configuration with petals as aerodynamic control surfaces are presented in Ref. [15]. In addition, the reentry-burn phase with up to three active engines was simulated in the Hypersonic Wind Tunnel Cologne (H2K) with cold gas. Results of these experiments as well as results of CFD simulations rebuilding these experiments can be found in Refs. [20, 21].

### Flight dynamics and GNC

Extensive mission analyses were carried out to assess the feasibility of the return mission for the different RETALT1 configurations, to identify the feasible domains that would enable the recovery, the associated propellant budget, and to support the identification of the mission and maneuverability needs, the mission and GNC requirements, and the sizing of the actuators. Flying qualities (FQ) analysis assessed the flight characteristics for the different phases, to assure the capability to perform a stable and controllable flight and recover the launcher. Eventually, the mission analysis and FQ led to the consolidation of the mission design for the scenarios considered (see Ref [10]).

To enable recovery and thus reusability it is critical to define a GNC solution able to guarantee a pinpoint landing for RETALT1. Compensation of the dispersions accumulated during the return flight is critical to achieve this objective. An ongoing activity is the development of novel Guidance solutions based on online trajectory optimization to achieve the required precision in an environment with large uncertainties. Optimization of the fuel consumption during the retro propulsion phase, while providing convergence guarantees, is also important to increase the feasibility of the return mission and the affordability of the recovery. State-of-the-art hybrid navigation technologies are being implemented for the reliable estimation of the vehicle states based on the fusion of inertial measurements together with GNSS (Global Navigation Satellite System) data (i.e., Galileo satellite constellation), together with radar altimeter information for the very last part of the landing (see Ref [22]). Multi-mode scheduled controllers will be designed and verified using modern robust control techniques, such as H-infinity and mu-synthesis/analysis.

The guidance for the landing approach is described in detail in Ref. [23].

### Structures

Structural concepts are being developed for both the landing structures and the Aerodynamic Control Surfaces (ACS). Key considerations in these studies are the derivation of appropriate load cases, including the thermal environment in close relation to the configuration and aerothermal investigations and the iteration on conception and sizing. Having a reuse lifecycle in the structures also makes reparability and maintainability important factors in the sizing that have to be balanced with the constraint to keep the mass for the structural components low.

The landing leg design predominantly makes use of carbon fiber reinforced plastics (CFRP) to minimize mass; however, some shielding is necessary to protect the main structural elements from the elevated temperatures during descent. The ACS makes use of both metals and CFRP to minimize mass, but due to the very high leading-edge temperatures during descent, a hybrid configuration is required.

Currently the detailed engineering of a demonstrator for a scaled landing leg for subsequent structural and stress tests is ongoing. First results of critical highly loaded components of the scaled landing leg are given in Ref. [12]. Furthermore, a first assessment of reaction forces of the full-scale model is done by simulation and different landing scenarios are investigated to proof if stable landing is feasible.

### Mechanisms and TVC

Mechanisms concepts are developed for the landing legs, control surfaces and thrust vector control system in the framework of the project. Important aspects of mechanism assessment and concept generation have been reliability and reusability as well as mass reduction, while keeping in line with current aerospace trends, considering electro-mechanical actuation where possible.

Landing leg and fin mechanism kinematics bring the stowed components into deployed/nominal position and lock them on their respective axes by passive means wherever possible. The selected landing leg configuration is composed of four legs with an inverted tripod configuration. The damping system is a combination of a reusable absorber and a crush cartridge. The fin mechanism is designed to be able to support large aerodynamic loads. The mechanisms and TVC are described in detail in Ref. [17].

### TPS developments

Due to high expected thermal loads during the re-entry phase of the two RETALT configurations, critical structural parts are covered with a cork Thermal Protection System (TPS). For reusability purposes, the TPS is designed in such a way, that it is easily replaceable and maintainable. Two processes where considered for this application, a sprayable and a trowelable solution. The TPS was enhanced in its material properties, formulation design and application process. A high-performance cork TPS with good thermal properties, flame resistance and good in situ applicability was developed as laid out in Ref. [24]. It was tested recently in the arc heated facilities at the Supersonic and Hypersonic Technologies Department of DLR in Cologne and showed very promising results.

## Conclusion

Substantial advances have been made in the technologies investigated in the RETALT project. Wind Tunnel Tests have been performed and evaluated, mission engineering design approaches have been implemented and a first loop of the development of a GNC concept has been performed. A structural and actuation demonstrator of an aerodynamic control surface has been built and landing legs have been designed in detail and will be tested soon. A novel TPS cork material has been developed for the use on reusable launcher first stages and has been tested. The reference configuration of RETALT1 has been expanded by adding a baseline configuration with planar fins to the petal concept and grid fins already in investigation. The reasoning for the change in the base line configuration has been laid out in detail in this paper.

In the last phase of the project detailed data evaluation and further testing will raise the TRL of the technologies investigated in the project to the expected level which is 5 for aerodynamics, aerothermodynamics, structures, mechanisms and TPS and 3 for the GNC concept. Furthermore, the exploitation possibilities of the technologies developed in the project will be assessed and evaluated regarding the needs of development and investigation still required to raise the TRL of the technologies to 8 or 9 and to implement the technologies in the European Launcher market.

## Abbreviations

ACS: Aerodynamic control surfaces
AEDB: Aerodynamic data base
ATDB: Aerothermodynamic data base
CFD: Computation fluid dynamics
CFRP: Carbon fiber reinforced plastics
DRL: Down range landing
GNC: Guidance navigation control
GNSS: Global navigation satellite system
GTO: Geo transfer orbit
H2K: Hypersonic wind tunnel cologne
LEO: Low earth orbit
MECO: Main engine cut-off
RTLS: Return to launch site
SSTO: Single stage to orbit
TMK: Trisonic wind tunnel cologne
TPS: Thermal protection system
TVC: Thrust vector control
CM: Pitch moment coefficient
Cp: Pressure coefficient
CoG: Center of gravity
AoA, Alpha: Angle of attack
*δ*: Deflection angle
